# Supplementary figures and images for: Towards a task to assess boredom-like states in pigs–Stimulus validation as a basis
Source: PLoS One. 2024 Oct 24;19(10):e0311843. doi: 10.1371/journal.pone.0311843 (PMC11501033; doi:10.1371/journal.pone.0311843)

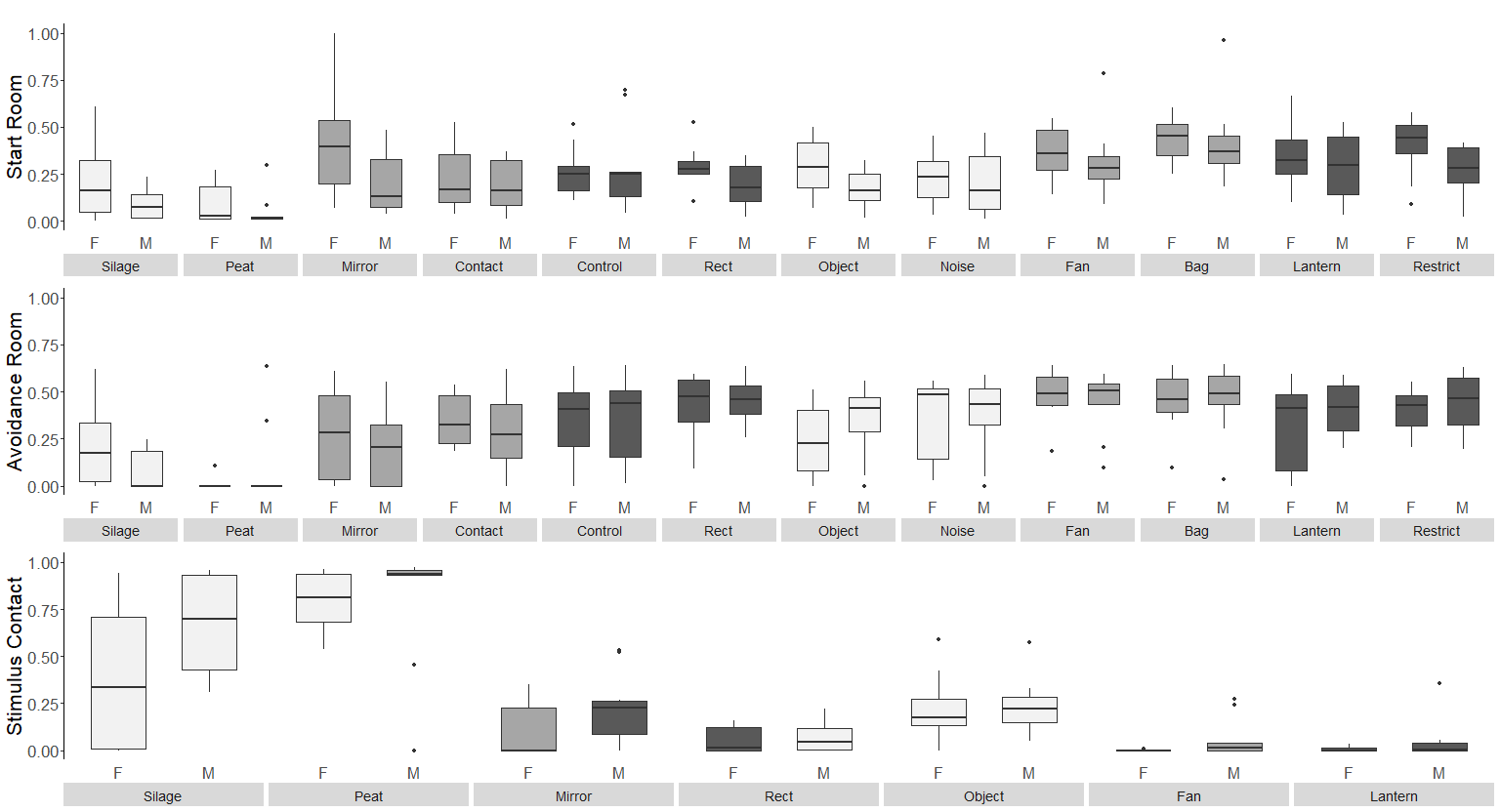

Supplement: S1 Fig — Boxes show medians and the lower as well as upper interquartile range. Whiskers represent 1.5 times the interquartile range. White boxes: Presumed positive stimuli, light grey boxes: Presumed ambiguous stimuli, dark grey boxes: Presumed negative stimuli. (TIFF) [file pone.0311843.s001.tiff]

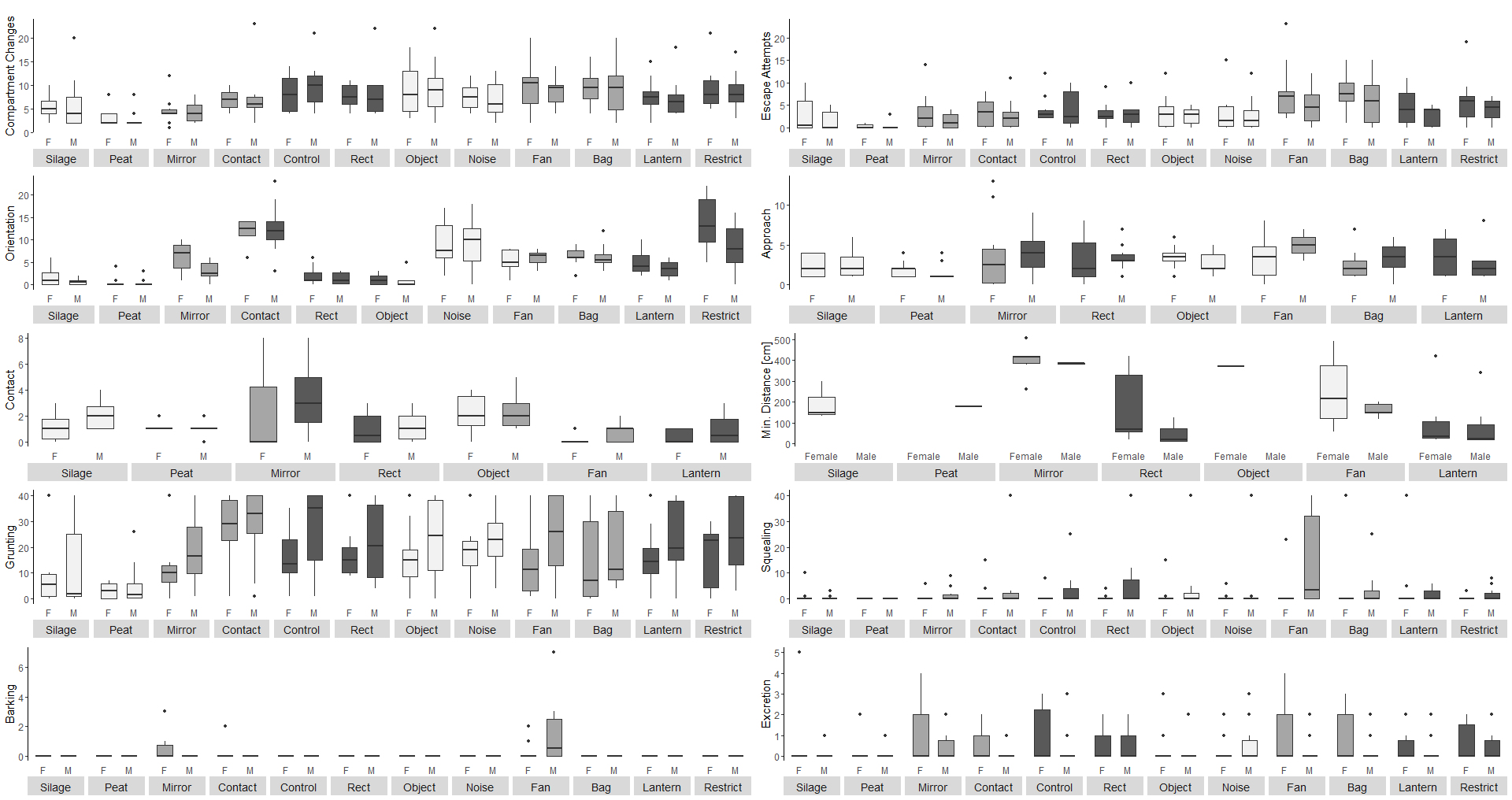

Supplement: S2 Fig — Boxes show medians and the lower as well as upper interquartile range. Whiskers represent 1.5 times the interquartile range. White boxes: Presumed positive stimuli, light grey boxes: Presumed ambiguous stimuli, dark grey boxes: Presumed negative stimuli. (TIFF) [file pone.0311843.s002.tiff]
