# Supplementary material for: Towards a task to assess boredom-like states in pigs–Stimulus validation as a basis
Source: PLoS One. 2024 Oct 24;19(10):e0311843. doi: 10.1371/journal.pone.0311843 (PMC11501033; doi:10.1371/journal.pone.0311843)
Supplement: Attachment — Submitted filename: Hintze et al_Responses to the reviewer.docx [file pone.0311843.s005.docx]

**Response letter 2**

to the reviewer who re-reviewed the manuscript “Towards a task to assess boredom-like states in pigs – stimulus validation as a basis” submitted to PLOS ONE by Sara Hintze, Heidi Heigl and Christoph Winckler

**Reviewer #2**: Thank you to the authors for their thorough responses to my previous comments, as well as for the corresponding revisions made in the manuscript. The additional details and rationale provided have greatly enhanced the clarity of this study and addressed my primary concerns.

In particular, I appreciate the improvements made to the completeness of reporting, which effectively demonstrate the value of this work. The use of the ARRIVE Guidelines and the inclusion of the ARRIVE Essential 10 Checklist were valuable additions and the updates to the text, figures, and methods ensure that the study can be better understood and replicated by future researchers. The additional information regarding the criteria for confirming pigs were habituated, as well as the updated plots on sex differences, made these aspects of the study much easier to interpret. These updates also offered reassurance that there were no significant differences between groups, which strengthens the findings.

The rationale for the selection of stimuli and outcomes is now clearer. While it remains somewhat surprising that the chosen stimuli and outcomes do not consistently facilitate comparison, this is now transparently communicated in a way that allows readers to better determine which stimuli might be suitable for comparison in future studies.

Thank you for your positive feedback and, especially, for highlighting which aspects of our revision you appreciate and why. Below we respond to your additional comments in blue.

I have only a few minor comments for this revised version of the manuscript:

• Line 528: Would it be possible to provide a value showing the space per pig in this study and compare it to other studies or requirements? For instance, providing evidence that these conditions were more likely to induce a negative state would strengthen the discussion.

Thank for you this thought. We are a bit surprised about this line of reasoning since you asked us in the first round of reviewing why animals were not kept in fully enriched conditions, but would like us now to strengthen the argumentation by stating that pigs were in a rather negative state (which was criticised before). We would still argue that we aimed to find a baseline by keeping pigs in as “neutral” conditions as somehow possible, thereby avoiding both “too good” and “too bad” conditions. With respect to the available space allowance, we have argued that pigs in our study had more space per pig than under conventional conditions, and that this higher space allowance in combination with a little amount of sawdust (piglets) and straw (gilts) was what we defined as “minimally enriched”.

Besides this, we could compare space allowances across studies, but we know that space allowance is only one of several factors affecting pigs’ affective states with especially enrichment being of paramount importance for this curios species that spends three quarters of their active time exploring, rooting and feeding. Thus, even though we would be very happy if we could provide more information on the pigs’ affective welfare by putting the conditions of our pigs into perspective, we don’t think that comparing space allowances will help with it.

• Adding a legend for the colors used in Figures 4 and 5 (box plots) would be valuable for clarity.

We agree with you and complemented the legends of Figures 4 and 5 accordingly: “White boxes: presumed positive stimuli, light grey boxes: presumed ambiguous stimuli, dark grey boxes: presumed negative stimuli.” We also added this information for the figures in the Supplementary Information.

• Line 248-249: It might be easier to interpret if you indicate that the control is the empty runway at the first mention. For example, “encompassed a Control (the empty runway), a green….”, or something similar

Thank you for this suggestion. We adapted the sentence, which now reads: “The four stimuli defined as potentially ambiguous (Fig 3F-K) encompassed a Control, i.e. the empty Runway, a green Rectangle, a green plastic Object, and an audio recording of Farm Noises.”
